# Association of Type 2 Diabetes and Hepatic Encephalopathy in Chronic Liver Disease Patients

**DOI:** 10.7759/cureus.17061

**Published:** 2021-08-10

**Authors:** Mohsin A Usmani, Attiya S Rahman, Qaiser Jamal, Muhammad Siddiqui

**Affiliations:** 1 Department of Medicine, Abbasi Shaheed Hospital & Karachi Medical and Dental College, Karachi, PAK; 2 Department of Research, Saskatchewan Health Authority, Regina, CAN

**Keywords:** diabetes mellitus type 2, severe hepatic encephalopathy, chronic liver disease, hepatitis c virus, metabolic disorder

## Abstract

Background

Hepatic encephalopathy (HE) is an important complication of hepatic cirrhosis and is an independent predictor of mortality in patients with cirrhosis. The prevalence of type 2 diabetes continues to increase at an alarming rate around the world, with even more people being affected by prediabetes. Diabetes leads to increased gastric transit and orocecal time, increased glutamase activity, and intestinal bacterial overgrowth, which may increase intestinal ammonia production. Thus, we speculated that diabetes mellitus (DM) might predispose cirrhotic patients to development and/or exacerbation of HE. The main purpose of this study is to determine the association of DM with severe HE in patients with chronic liver disease (CLD).

Methods

This case-control study (122 cases and 122 controls) was conducted for 6 months on patients who fulfilled the inclusion criteria and were selected from the Medical department, Abbasi Shaheed Hospital, Karachi, after taking informed consent. Demographic data were presented as simple descriptive statistics giving mean and standard deviation and qualitative variables were presented as frequency and percentages. Chi-square was applied and the odds ratio (OR) was calculated taking a p-value of ≤ 0.05 as statistically significant.

Results

Out of a total of 244 patients, 122 patients had CLD with DM (case group) and 122 participants had CLD without DM (control group). The mean and standard deviation of age in the case and control groups in our study was 43.29±3.79 and 45.49±5.40. The mean and standard deviation of the duration of disease in the case and control groups in our study was 3.18±1.22 and 3.72±1.36. Males were 53 (43.44%) and 56 (45.10%) in the case and control groups, whereas females were 69 (56.56%) and 66 (54.10%) in the case and control groups, respectively. Out of 122 patients in the case group, 73 (59.84%) and 49 (40.16%) patients developed and did not develop severe HE, respectively. Out of 122 patients in the control group, 50 (40.98%) and 72 (59.02%) patients developed and did not develop severe HE, respectively. Binary logistic regression analysis showed an association of severe HE with DM (p-value: 0.93, OR: 1.033, 95% CI: 0.586-1.599).

Conclusion

This study demonstrates that HE is a common occurrence in CLD patients. There was not a direct relationship of DM with the severity of HE was observed. However, further research with larger sample size and involving a multicenter setting is warranted.

## Introduction

Type 2 diabetes mellitus (DM) is a risk factor for chronic liver disease (CLD), and approximately 30% of patients having cirrhosis of the liver go on to develop diabetes. Cirrhotic and non-cirrhotic hepatitis C virus liver infection has been associated with DM; this can aggravate the course of the liver infection and can induce a lower sustained response to antiviral treatment. It has been found by fewer studies that diabetes increases the risk of liver complications and death in patients with cirrhosis. However, treatment of diabetes in these patients is complex, as antidiabetic drugs can aggravate hypoglycemic episodes and lactic acidosis [1]. Patients with CLD and DM are increasing in number. With the global epidemics of obesity and nonalcoholic fatty liver disease, treatment options should be evaluated essentially [2].

Hepatic encephalopathy (HE) is a neuropsychiatric syndrome associated with hepatocellular failure. It is a cognitive dysfunction in cirrhotic patients associated with impaired prognosis and accounts for nearly 20℅ cases per year in patients with cirrhosis and 30%-45℅ can develop overt encephalopathy at any time [3]. HE is a reversible metabolic encephalopathy with multifactorial pathogenesis [4]. The widely accepted hypothesis is that encephalopathy is due to a failure of hepatic clearance of gut-derived toxins, although ammonia remains the toxin of interest [5]. HE is characterized by personality changes, intellectual impairment, and a depressed level of consciousness. An important prerequisite for the syndrome is a diversion of portal blood into the systemic circulation through portosystemic collateral vessels [6].

DM is a chronic metabolic disorder that has emerged as a great socioeconomic burden for the developing and developed world. The past two decades have witnessed a significant increase in the prevalence of this devastating illness [7, 8]. Hyperammonemia plus inflammatory response plays a crucial role in HE. DM and insulin resistance are characterized by releasing and enhancing these pro-inflammatory cytokines and additionally, have been related to HE [9]. Patients with diabetes showed an increased risk of overt HE in comparison with non-cirrhotics. Type 2 DM could impair HE by different mechanisms that include: a) increasing glutaminase activity; b) impairing gut motility and promoting constipation, intestinal bacterial overgrowth, and bacterial translocation [10]. The main purpose of this study was to determine the association of DM with severe HE in patients with CLD.

## Materials and methods

This case-control study with non-probability consecutive sampling was conducted at the Department of Medicine, Abbasi Shaheed Hospital, Karachi, for a period of six months. The sample size was calculated by taking 95% two-sided significance level (1-alpha), 90% power 58.5% in cases (severe HE in diabetics), and 42.6% in control (severe HE in non-diabetics). Total 244 patients were enrolled in the study; 122 patients with DM (cases) and 122 patients without DM (controls). The required sample size was calculated using the World Health Organization sample size calculator.

Inclusion criteria

● CLD patients for more than one-year duration.

● CLD patients with DM labeled as case and without DM labeled as control

●Type II diabetes duration more than 10 years.

● Age 20-60 years

Exclusion criteria

● Non-consenting patients

● Critically ill patients

● Patients with a history of psychiatric illness.

● Patients taking sedatives or anxiolytics

● Patients with a history of congestive cardiac failure

● Patients with a history of post-myocardial infarction

● Pregnancy

● Patients with a history of chronic renal failure

● Patients with a history of hepatocellular carcinoma

Operational definition

Chronic Liver Disease

Patients presenting with any three or more of the following were labeled as suffering from CLD:

● Shrunken liver size, i.e., longitudinal diameter of the right and the left lobes <90 mm and 70 mm, respectively, on ultrasound scan of the abdomen.

● Nodularity of liver surface assessed by ultrasound abdomen.

● Coarsening of liver echotexture assessed by ultrasound abdomen.

● Ascites detected by ultrasound abdomen.

● Portal hypertension, i.e., enlarged portal vein size more than 13mm was measured as the largest anterior-posterior diameter at the crossing point with the hepatic artery assessed by ultrasound abdomen.

Severe HE

Patients were labeled as having severe HE by a consultant physician according to West Haven Criteria ≥3 in which somnolence to semi stupor, responsive to stimuli, confusion, gross disorientation, bizarre behavior, or coma [11].

Data collection procedure

This study was conducted after permission from the institutional ethical review committee was taken prior to the commencement of the study. Patients who fulfill the inclusion criteria were selected from the Department of Medicine, Abbasi Shaheed Hospital, Karachi, after taking informed consent. CLD patients with DM were labeled as (Case) and CLD patients without DM were taken as (Control). A brief history of the duration of the disease was taken. Patients admitted with HE were evaluated by the researcher and experienced clinical supervisor with more than five years of experience based on West Haven Criteria; all patients with West Haven Criteria ≥3 were included in the study. The findings of the outcome of HE or non-HE were collected.

Data analysis procedure

Data were analyzed by SPSS version 16.0. Mean and standard deviation was calculated for age and duration of CLD. Frequency and percentages were calculated for gender, Child-Pugh score (A/B/C), and severe HE (yes/no). Chi-square was applied to compare both groups taking a p-value of ≤ 0.05 as significant. The odds ratio (OR) was calculated to see the association between case and control. Confounders were controlled through stratification of age, gender, Child-Pugh score (A/B/C), and duration of CLD to see the effect of these on the outcome variable. The post-stratification chi-square test was applied to take a p-value of ≤ 0.05 as statistically significant.

## Results

A total of 244 patients participated in the study; of which 122 were in the case group and 122 were in the control group. The mean age in the case group was 43.29±3.79 and in the control group was 45.49±5.40years ranged from 29 to 58 years. Out of 122 patients in both case and control groups, the minimum duration of disease was 1 while the maximum duration of disease was five years. The mean duration of disease in the case group was 3.18±1.22 years and in the control group was 3.72±1.36 years (Table 1).

**Table 1:** Descriptive statistics of age and duration of disease in case and control (122 case and 122 control); n=244

Variable	Mean±SD	Standard Deviation	Min-Max
Age case (years)	43.29	±3.79	29-58
Age control (years)	45.49	±5.40	29-58
Duration of disease case (years)	3.18	±1.22	1-5
Duration of disease control (years)	3.72	±1.36	1-5

Among type II diabetics, a total of 73 (59.83%) develop and 49 (40.16%) did not develop severe HE. Similarly, among non-diabetics 50 (40.98%) develop and 72 (59.01%) did not develop severe HE; (p-value <0.001). However, study results showed no significant association was observed between DM and prevalence of HE (OR: 1.033; 95% CI: 0.586-1.599; p-value: 0.93). No significant association was detected between age and severe HE (Table 2) in both the case group (p-value 0.52) and the control group (p-value 0.32).

**Table 2:** Severe hepatic encephalopathy of case and control according to age (122 case and 122 control); n=244

Age (years)	Severe Hepatic Encephalopathy Case		Total	Severe Hepatic Encephalopathy Control		Total
	Yes	No		Yes	No	
20-40	50 (68.5%)	33 (67.3%)	83 (68%)	23 (46%)	29 (40.3%)	52 (42.6%)
41-60	23 (31.5%)	16 (32.7%)	39 (32%)	27 (54%)	43 (59.7%)	70 (57.4%)
Total	73 (100%)	49 (100%)	122 (100%)	50 (100%)	72 (100%)	122 (100%)
P-value	0.52			0.32		

The summary of gender and severe HE is presented in Table 3. Nearly two-thirds of females (n=34; 69.4%) and 15 (30.6%) males had no severe HE in the case group. A significant association (p-value 0.01) was observed between gender and severe HE in the case group. Similarly, a significant association (p-value 0.02) was seen between gender and severe HE in the control group.

**Table 3:** Severe hepatic encephalopathy of case and control according to gender (122 case and 122 control); n=244

Gender	Severe Hepatic Encephalopathy Case		Total	Severe Hepatic Encephalopathy Control		Total
	Yes	No		Yes	No	
Male	38 (52%)	15 (30.6%)	53 (43.4%)	29 (58%)	27 (37.5%)	56 (45.9%)
Female	35 (48%)	34 (69.4%)	69 (56.6%)	21 (42%)	45 (62.5%)	66 (54.1%)
Total	73 (100%)	49 (100%)	122 (100%)	50 (100%)	72 (100%)	122 (100%)
P-value	0.01			0.02		

Classification for the duration of disease with respect to severe HE in the case and control groups showed in Table 4. A total of 17 (23.3%) patients with 1-2 years, 30 (41.1%) patients with 3-4 years, and 26 (35.6%) with 5-6 years developed severe HE in the case group. A significant association (p-value 0.04) was observed between the duration of disease and severe HE in the case group. However, no significant association (p-value 0.20) was observed between the duration of disease and severe HE in the control group.

**Table 4:** Severe hepatic encephalopathy of case and control according to the duration of disease (122 case and 122 control); n=244

Duration of Disease	Severe Hepatic Encephalopathy Case		Total	Severe Hepatic Encephalopathy Control		Total
	Yes	No		Yes	No	
1-2 years	17 (23.3%)	21 (42.9%)	38 (31.2%)	21 (42%)	34 (47.2%)	55 (45.1%)
3-4 years	30 (41.1%)	23 (46.9%)	53 (43.4%)	14 (28%)	26 (36.1%)	40 (32.8%)
5-6 years	26 (35.6%)	05 (10.2%)	31 (25.4%)	15 (30%)	12 (16.7%)	27 (22.1%)
Total	73 (100%)	49 (100%)	122 (100%)	50 (100%)	72 (100%)	122 (100%)
P-value	0.04			0.20		

Categorization for Child-Pugh score with respect to severe HE in case group 46.6%, 23.3%, and 30.1% was in the Child-Pugh score groups A, B, and C, respectively, developed severe HE (Table 5). In both case (p-value 0.02) and control (p-value<0.001) groups, a significant association was observed between Child-Pugh score and severe HE.

**Table 5:** Severe hepatic encephalopathy of case and control according to Child-Pugh score (122 case and 122 control); n=244

Child-Pugh Score	Severe Hepatic Encephalopathy Case		Total	Severe Hepatic Encephalopathy Control		Total
	Yes	No		Yes	No	
A	34 (46.6%)	29 (59.2%)	63 (51.6%)	26 (52%)	27 (37.5%)	53 (43.4%)
B	17 (23.3%)	18 (36.7%)	35 (28.7%)	21 (42%)	22 (30.5%)	43 (35.2%)
C	22 (30.1%)	02 (4.1%)	24 (19.7%)	03 (6%)	23 (32%)	26 (21.4%)
Total	73 (100%)	49 (100%)	122 (100%)	50 (100%)	72 (100%)	122 (100%)
P-value	0.02			<0.001		

## Discussion

HE represents a challenging clinical complication of liver insufficiency and presents with a wide spectrum of neuropsychiatric symptoms that range from mild disturbances in cognitive function to coma to even death. Our study showed that out of a total of 244 patients, 122 patients had CLD with DM (Case Group) and 122 participants had CLD without DM (Control Group).

Sigal et al. found that 54 patients (83%) had HE (33 mild, 21 severe). Twenty patients (31%) had DM. HE was present in 19 (95%) patients with diabetes and 35 (78%) patients without diabetes (p-value 0.087). The severity of HE was greater in diabetic (35% mild, 60% severe) than in nondiabetic patients (58% mild, 20% severe) (p-value 0.007). In both the mild and severe HE categories, the severity of liver disease in diabetic patients was otherwise milder than in the nondiabetic patients [10]. Another study results revealed 50.3% HE of which 118 patients had DM (33.5%). Patients with DM had a significantly higher prevalence (58.5% vs 42.6%; p-value 0.03) and severity of HE (p[trend] = 0.01) than patients without DM. However, there were no significant differences between the two groups in terms of Child-Pugh class, MELD scores, the presence of ascites, and esophageal varices. Patients with DM had higher platelet counts than those without DM (p[trend] = 0.003). In age and gender subgroup analyses, older patients and men with DM had significantly greater evidence of HE (p-value 0.02 and 0.03, respectively). Multivariate analysis showed that DM (p-value 0.03) and older age (p-value 0.006) were independently related to HE [12], as increased transient time secondary to autonomic dysfunction in diabetes that often occurs in elderly patients may lead to HE [13], whereas the association of gender was non-significant [12].

Jepsen et al. study included 862 patients of whom 193 (22%) had diabetes. In total, they experienced 115 first-time episodes of overt HE during the follow-up. Fewer diabetics than non-diabetic patients were in Child-Pugh class C at baseline (13% vs 23%), yet they had a higher cumulative risk of first-time overt HE (26.0% vs 15.8% after one year), and their episodes of first-time overt HE were more likely to progress beyond grade 2 (64% vs 42% of episodes progressed to grade 3 or 4, p-value 0.01 for independence between diabetes and highest HE grade). After the confounder adjustment, the hazard ratio of first-time overt HE for diabetics versus non-diabetic patients was 1.86 (95% CI: 1.20-2.87) [14].

Xiaochun et al. found out of the 436 patients who underwent TIPS, 85 (19.5%) had diabetes at admission and 126 (28.9%) had HE after TIPS. Patients with DM more frequently had HE compared with those without DM (44.7 vs 25.1%; p-value<0.001). The logistic regression analysis showed that DM (p-value 0.015) and age (p-value 0.002) were independent risk factors for HE after TIPS. Finally, using the Kaplan-Meier curves, we found that diabetes significantly increases the incidence of overt HE (log-rank p-value 0.026) [15]. Shaheen et al. found patients with cirrhosis with long-standing and uncontrolled DM are more likely to develop HE. Autonomic neuropathy, which may complicate patients with cirrhosis with long-standing uncontrolled diabetes, may play a role in the pathogenesis of HE in these patients [16].

Ghada et al. found diabetic patients had a higher frequency of all grades of HE mean number of attacks for each patient in the past three months is 1.9±0.3 versus 0.8±0.1 in non-diabetics with unclear precipitating factor in 43% of diabetic patients versus 23% in non-diabetic patients. Patients on oral hypoglycemic drugs represented 14.3% of diabetic patients. Patients with HbA1c > 11% were 43% among patients on oral hypoglycemic drugs versus 23% with insulin. Patients on oral hypoglycemic drugs had a higher frequency of hepatic coma. The mean number of attacks experienced by each patient rises with increased concentration of HbA1c from 0.8±0.2 at level <7% to 6.4±3 for level > 11%. The mean number of attacks increased with the duration of diabetes from 1±0.4 for 15 years [17].

Yi-Wen et al. proved that chronic hepatitis B and chronic hepatitis C patients with newly diagnosed DM were more likely to develop liver cirrhosis and further decompensation. As a consequence, the scenario regarding DM increased HE risk in cirrhotic patients seemed to be the rationale for the story to be continued while one may have passed the point of no return on the track to liver failure [18]. Ampuero et al. found patients with diabetes showed raised risk of overt HE in comparison with non-cirrhotics. Type 2 DM could impair HE by different mechanisms that include: a) increasing glutaminase activity; b) impairing gut motility and promoting constipation, intestinal bacterial overgrowth, and bacterial translocation. Despite insufficient clarity about the practicability of anti-diabetic therapy and the most efficacious therapy, we would have to pay special attention to the management of type 2 DM and insulin resistance in cirrhotic patients [9].

Arshad et al. found HE was present in 50.3% of patients at the time of admission and 33.5% of patients were diabetic. Chronic hepatitis C was the most common cause of cirrhosis (71.6%). HE at admission was present in 58.5% of diabetics and 42.6% of non-diabetics (p-value 0.03). The severity of HE was higher in patients with diabetes than those without diabetes (p-value for trend 0.01)[19]. In another study, 240 patients with liver cirrhosis participated and were observed for a median of 17 months. At study inclusion, DM was present in 65 patients (27.1%) and covert HE was identified in 33.3%. A more preserved liver function was found in patients with DM versus those without DM (model of end-stage liver disease 9 vs 10). Overall, a higher risk for the presence of covert HE and the occurrence of overt HE may be observed in correlation with DM in patients with liver cirrhosis [20].

El Soud et al. found out on 100 cirrhotic patients with DM (50 encephalopathic patients [group I] and 50 non-encephalopathic patients). Cirrhotic patients with longstanding and uncontrolled DM are more likely to have higher grades of HE. Autonomic neuropathy, which may complicate cirrhotic patients with longstanding uncontrolled diabetes, may play a role in the pathogenesis of HE in these patients [21]. Identifying DM at an early stage and addressing it will reduce morbidity and mortality of cirrhotic patients presenting with HE and improve their quality of life.

## Conclusions

This study demonstrates that HE is a common occurrence in CLD patients. There was no direct relationship was observed in DM with the severity of HE. However, there is still a great deal about severe HE that is unknown. Recognition of HE will depend on a high level of awareness for this syndrome and potentially will help in better management of patients with CLD. Further research with larger sample size and involving a multicenter setting is warranted.
